# Effects of Treatment With Hypnotics on Reduced Sleep Duration and Behavior Abnormalities in a Mouse Model of Fragile X Syndrome

**DOI:** 10.3389/fnins.2022.811528

**Published:** 2022-06-02

**Authors:** Rachel Michelle Saré, Abigail Lemons, Carolyn Beebe Smith

**Affiliations:** Section on Neuroadaptation and Protein Metabolism, Department of Health and Human Services, National Institute of Mental Health, National Institutes of Health, Bethesda, MD, United States

**Keywords:** fragile X, hypnotics, sleep, social behavior, activity, anxiety-like behavior

## Abstract

Many patients with fragile X syndrome (FXS) have sleep disturbances, and *Fmr1* knockout (KO) mice (a model of FXS) have reduced sleep duration compared to wild type (WT). Sleep is important for brain development, and chronic sleep restriction during development has long-lasting behavioral effects in WT mice. We hypothesized that the sleep abnormalities in FXS may contribute to behavioral impairments and that increasing sleep duration might improve behavior. We treated adult male *Fmr1* KO and WT mice subacutely with three different classes of hypnotics (DORA-22, ramelteon, and zolpidem) and caffeine, a methylxanthine stimulant, and we tested the effects of treatments on sleep duration and behavior. Behavior tests included activity response to a novel environment, anxiety-like behavior, and social behavior. As expected, all hypnotics increased, and caffeine decreased sleep duration in the circadian phase in which drugs were administered. Caffeine and DORA-22 treatment significantly reduced activity in the open field regardless of genotype. Other effects were not as apparent.

## Significance Statement

We tested the efficacy of subacute hypnotic treatment for ameliorating behavioral symptoms in a mouse model of fragile X syndrome. As expected, all hypnotics significantly increased sleep duration in the circadian phase in which they were administered. Conversely, caffeine treatment reduced sleep during the phase in which it was administered. Both caffeine and DORA-22 reduced activity in the open field; but effects on social behaviors were minimal.

## Introduction

Sleep is thought to play a key role in brain development and associated synaptic plasticity, and abnormal sleep may be involved in the unfolding of neurodevelopmental disorders (Picchioni et al., 2014). People with neurodevelopmental disorders have a high prevalence of sleep abnormalities which are correlated with the severity of behavioral impairments (Picchioni et al., 2014). Furthermore, in a recent study, we found an association between diagnosed sleep problems and scores on a questionnaire indicative of social communication deficits in non-diagnosed siblings of patients with autism (Saré and Smith, 2020), a population susceptible to developing autism.

Studies in experimental animals provide insights into the critical nature of sleep during development. In rats, early postnatal REM sleep-deprivation resulted in long-term changes that became manifest in adulthood, including decreased brain weight and increased levels of anxiety-like behavior and hyperactivity (Mirmiran et al., 1983). Additionally, chronic sleep restriction beginning early in postnatal development in wild-type (WT) mice resulted in behavioral changes, particularly in social behavior and activity levels. The behavioral changes persisted even after recovery sleep (Saré et al., 2015, 2019).

In the present study, we focused on the role of disordered sleep in the neurodevelopmental disorder, fragile X syndrome (FXS). In FXS, the gene *FMR1* is silenced due to a CGG repeat expansion and its protein product, Fragile X Mental Retardation Protein (FMRP) is absent. FXS is characterized by intellectual impairment and is often associated with social behavior abnormalities. Many people with FXS also have sleep abnormalities (Picchioni et al., 2014), and the presence of sleep abnormalities correlates with diminished ability to pay attention, difficulty in social interactions, and resistance to change (Kronk et al., 2010). Available evidence suggests that the prevalence of sleep abnormalities may even be underestimated in this population. In a questionnaire study, a significantly higher proportion of participants with FXS reported receiving medicine to help sleep than reported having sleep problems (Kronk et al., 2010). Animal studies show that FMRP is important for both circadian rhythm and sleep. The *Drosophila* model of FXS (*dfmr1)* has both abnormal sleep and circadian rhythm disruption, likely due to the fact that *Drosophila* not only lacks FMRP but also FXR2, a homolog of *Fmr1* not found in *Drosophila* (Dockendorff et al., 2002; Bushey et al., 2009). In mammalian models, the combination of deleting *Fmr1* and related homolog *Fxr2* resulted in complete circadian rhythm disruption (Zhang et al., 2008). Even deletion of *Fmr1* alone resulted in a slightly shorter period length demonstrated by behavior in constant darkness (Zhang et al., 2008). Furthermore, deletion of *Fmr1* alone resulted in significantly reduced sleep duration in mice compared to WT mice (Saré et al., 2017a; Boone et al., 2018), and this was exacerbated by the heterozygous deletion of *Fxr2* (Saré et al., 2017a).

*Fmr1* knockout (KO) mice recapitulate many features of the human disease, including behavioral abnormalities. Of note, *Fmr1* KO mice demonstrate social abnormalities, decreased levels of anxiety-like behavior, hyperactivity, and learning and memory deficits (Qin et al., 2002, 2015a,b; Liu et al., 2011; Saré et al., 2016, 2017b). We hypothesized that increasing sleep duration may improve behavior in *Fmr1* KO mice. In this study, we treated *Fmr*1 KO mice subacutely with three different classes of hypnotics and recorded effects on sleep duration, open-field activity, anxiety-like behavior, and social behavior. The three hypnotics used act *via* different mechanisms. Zolpidem is a nonbenzodiazepine hypnotic acting through the GABA_*A*_ receptor and is a widely prescribed prescription medication for insomnia (Depoortere et al., 1986). Ramelteon is a melatonin receptor agonist FDA-approved for insomnia in 2005, it is efficacious for producing increased nonREM sleep in rodents (Fisher et al., 2008). Dual orexin receptor antagonists (DORAs) have also received FDA approval (in 2014) for the treatment of insomnia and do not have lingering sedative effects or lead to subsequent cognitive defects in animal models following administration (Michelson et al., 2014).

We found that all three classes of hypnotics significantly increased sleep duration in the inactive phase (when the animals were treated). Conversely, caffeine, a stimulant significantly decreased sleep duration in the inactive phase. Testing in the active phase showed that both DORA-22 and caffeine resulted in significantly decreased activity in the open field regardless of genotype. Behavioral differences in anxiety-like behavior and social behavior were less apparent. Our data indicate that treatment with hypnotics for 1-week improved sleep duration in both WT and *Fmr1* KO mice, but effects on behavior tests were minimal.

## Materials and Methods

### Animals

These studies were performed on male *Fmr1* KO mice (C57BL/6J-Fmr1^tm1Cgr^ Jackson Strain: 003025) generated in house by heterozygous female and WT male breeding pairs. Animals were maintained in a central facility with access to food and water *ad libitum*. Pups were weaned between 21 and 23 days of age and were genotyped by means of PCR amplification of DNA from tail snips as previously described (Qin et al., 2005). Mice were group housed until treatment began. From birth to 40 ± 7 days of age, animals were maintained on a standard 12:12 light/dark cycle (with lights on at 6:00 a.m.). At 40 ± 7 days of age, and throughout behavior testing, animals were shifted to an altered 12:12 light/dark (with lights on at 1:00 p.m.). All procedures were approved by the National Institute of Mental Health Animal Care and Use Committee and were conducted according with the National Institutes of Health Guidelines on the Care and Use of Animals.

### Drug Treatments

**FIGURE 1:**
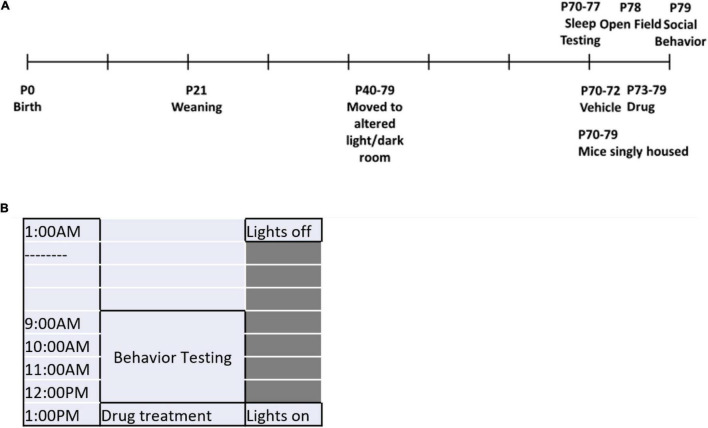
Experimental timeline. **(A)** The experimental timeline is shown. Mice were moved to the holding room under an altered light/dark cycle beginning at P40 and remained there for the duration of the testing. At P70, mice began sleep testing and the treatment regimen simultaneously (during which time they were also singly housed). From P70-P72, treatment for all mice was the vehicle. At P73-P79, mice were switched to their respective drug treatment groups. Sleep testing ended at P77. Open field testing was conducted on P78 and social behavior testing was conducted on P79. **(B)** The timeline for behavior testing days is shown. Lights were turned off at 1:00 a.m. Behavior testing occurred from 9:00 a.m. to 1:00 p.m. Hypnotic administration was given at 1:00 p.m. and lights were turned on.

We administered drugs orally to prevent the demonstrated effects of injections themselves on sleep duration (Lemons et al., 2018). We used peanut butter balls (equal mix of ground mouse chow and peanut butter) as the vehicle for drug administration. Peanut butter balls were approximately 1.2 g and were consumed within 5 min by each mouse. Vehicle treatment was ground mouse chow and peanut butter. Drugs were mixed into the ground mouse chow in the appropriate dose as follows: Caffeine (10 mg/kg) (Tocris, Minneapolis, MN), DORA-22 (30 mg/kg) (Merck, Kenilworth, NJ), Ramelteon (10 mg/kg) (Takeda, Deerfield, IL), and Zolpidem (10 mg/kg) (Tocris). The doses of hypnotics used were based on previous studies in rodents to promote sleep. The half-life in rodents of caffeine is 40–60 min (Hartmann and Czok, 1980), DORA-22 is 37.8 min (Gotter et al., 2013), ramelteon is less than 60 min (Yoshimoto et al., 2021), and zolpidem is about 20 min (Garrigou-Gadenne et al., 1989). Drug treatments were assigned in a paired design (one WT and one *Fmr1* KO mouse per treatment group per study group). Eight mice were studied in each cohort, representing four out of the five treatment groups. We alternated which treatment group was not represented in each cohort to get an even distribution across the experiment. As much as possible, litters were kept together in the study, but mice of the same genotype in a litter were not given the same drug. Treatment was administered daily throughout the study (Figure 1) at 1:00 p.m. The experimenter was blind to genotype but administered treatments and conducted behavioral tests. Most behavioral measures were assessed with automated equipment and would not be sensitive to bias.

### Timeline

Treatment commenced at 70 ± 7 days of age. Beginning on Day 1 of the treatment, mice were singly housed for the duration of the study. Single-housing was necessary because of home-cage monitoring for sleep duration. Bedding was not changed during treatment to obviate the effect of cage-changes on sleep (Saré et al., 2018). From Days 1 to 3, mice were treated with vehicle to habituate to consuming the peanut butter ball. On Day 4, mice were switched to their respective drug treatment for the remainder of the study. Sleep duration in the active and inactive phases was recorded from Days 1 to 7. Treatment continued as mice underwent open field testing and social behavior testing each test separated by 1 day. These behavior tests were conducted between 9 a.m. and 1 p.m. in the dark during the active phase. Although behavior testing was conducted by multiple experimenters, all experimenters were female so as to decrease potential variability (Sorge et al., 2014).

### Home-Cage Monitoring

We measured sleep duration before and after drug treatment by means of the home-cage monitoring system (Columbus Instruments, Columbus, OH). Movement was detected by photobeams and 40 consecutive seconds of inactivity was considered a bout of sleep as has been previously validated (Pack et al., 2007).

### Open Field Activity

To assess activity and anxiety-like behavior, mice were placed in a novel open field arena (Coulbourn Instruments, Whitehall, PA) for 30 min, and horizontal movements were recorded in 5 min epochs over 30 min as previously described (Saré et al., 2016). The total horizontal distance traveled was recorded as a measure of activity, and the ratio of distance traveled in the center to total distance traveled was an inverse measure of anxiety-like behavior. Although all animals were placed in the open-field chambers, there were occasional errors that prevented data collection.

### Social Behavior

To assess social behavior, we used the standard three-chambered apparatus (Nadler et al., 2004). The test consisted of three phases. (1) Habituation: mice explored the empty chamber for 5 min. If a mouse spent more than 3 min in any chamber, it was eliminated from the study. (2) Sociability: a stranger mouse (age- and sex-matched) was placed in a social enclosure (inverted wire cup) in one chamber and an empty enclosure was placed in the opposite chamber. The test mouse explored for 5 min. (3) Preference for social novelty: a novel stranger mouse was placed in the previously empty social enclosure and the test mouse explored for 5 min. The time spent in each chamber was detected by photobeams. The time spent actively sniffing each mouse/object was recorded by an experimenter during the experiment. The enclosure was illuminated by a red light.

### Statistical Analysis

After data collection, we determined animals that were outliers for low weight (runts). One WT mouse was determined to be a runt and was eliminated from the study. Following removal of the runt, we determined statistical outliers (two standard deviations from the mean) in each of the behavioral categories. For open field, all open field data were removed for an animal that had two datapoints that reached statistical outlier criteria. For social behavior and sleep duration, data were not included if one measure reached statistical outlier criteria. Importantly, only the behavioral domain for which we noted odd behavior (as determined by the statistical outlier criteria) was removed; the other behavioral domains were still analyzed.

The resulting data were analyzed by means of a mixed model ANOVA (Huynh-Feldt-corrected for lack of sphericity) with genotype and treatment as between subject variables. Since caffeine and the hypnotics have opposite effects on sleep, their effects were analyzed separately. The vehicle group was the same for both analyses. Within subject variables were Day (sleep), epoch (open field), and chamber (social behavior). When appropriate, Bonferroni-corrected *post-hoc t*-tests were applied. Degrees of freedom with sphericity assumed are reported.

## Results

### Body Weights

Body weights were well matched among groups with the average of all included animals at the start of enrollment being 27.45 ± 2.11 g (mean ± SD) (Supplementary Table 1).

### Sleep Duration

**FIGURE 2:**
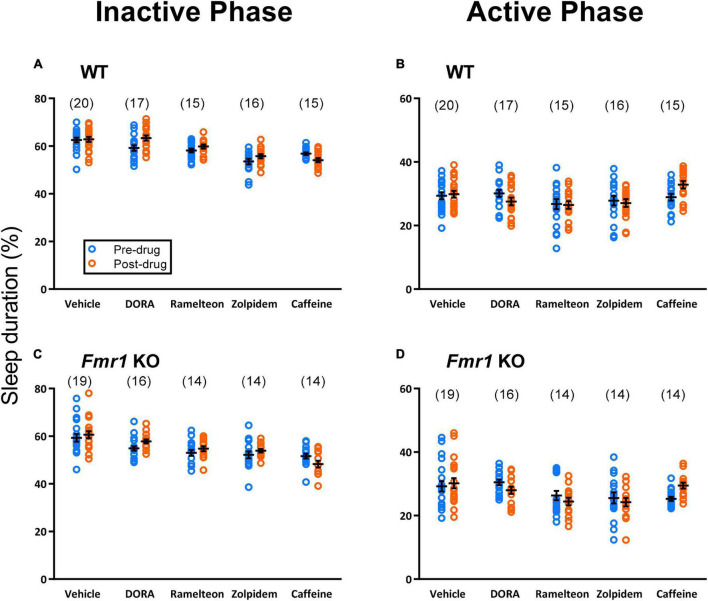
Sleep duration was analyzed employing a within subject design. Sleep duration on Day 3 (pre-drug) was compared with the average sleep duration for Days 4–5 (post-drug). Each point represents the value for a single animal. Lines represent means ± SEM for the number of animals indicated in parentheses. Sleep duration in the inactive phase **(A,C)** showed a statistically significant treatment × day interaction for both the hypnotics (*p* < 0.001) and caffeine analyses (*p* < 0.001). *Post-hoc t*-tests showed that, regardless of genotype, DORA-22 (7 ± 1% (mean ± SEM of individual percent changes), *p* < 0.001), ramelteon (4 ± 1%, *p* = 0.003), and zolpidem (4 ± 1%, *p* = 0.001) significantly increased sleep duration compared to pre-drug. Caffeine significantly decreased sleep duration compared to pre-drug (−5 ± 2%, *p* < 0.001). Sleep duration in the active phase **(B,D)** also showed a significant treatment × day interaction for both the hypnotics (*p* < 0.001) and caffeine analyses (*p* = 0.008). P*ost-hoc t*-tests showed that DORA-22 (−8 ± 2%, *p* < 0.001) and zolpidem (−3 ± 3%, *p* = 0.046) significantly decreased sleep duration in the active phase, whereas caffeine (17 ± 2%, *p* < 0.001) significantly increased sleep duration in the active phase. Ramelteon tended to decrease sleep duration in the active phase (−2 ± 3%, *p* = 0.096).

We examined sleep duration in both the active (dark) and inactive (light) phases (Figure 2). All animals received vehicle treatment for 3 days before being transferred to their respective drug condition, allowing us to employ a within subject design for the effect of drug on sleep duration.

Following 2 days of habituation to single housing, we analyzed Day 3 sleep duration (vehicle-treatment for all animals) as the control condition. We compared it to the average duration on Days 4–5 (assigned drug condition) to determine the effect of the drug. Statistical analysis was performed by means of mixed model ANOVA with day (Day 3 vs. mean of Days 4–5) as a within subject variable and genotype (WT, *Fmr1* KO) and treatment (vehicle, DORA-22, ramelteon, zolpidem) as between subject variables. For the analysis of effects of caffeine, we also used a mixed model ANOVA with day (Day 3 vs. Days 4–5) as a within subject variable and genotype (WT, *Fmr1* KO) and treatment (vehicle, caffeine) as between subject variables. We analyzed the inactive phase (in which animals received the treatment) and active phases separately.

In the inactive phase, the treatment × day interactions for both hypnotic and caffeine treatments were statistically significant (*p* < 0.001) (Tables 1, 2) indicating that, regardless of genotype, effects of drug treatments on sleep duration were significantly different from vehicle. We further analyzed for drug-specific effects by means of Bonferroni-corrected *post-hoc t*-tests and found statistically significant differences between Day 3 and mean of Days 4–5 for all drug treatments, but not for vehicle. DORA-22 (7 ± 1% (mean ± SEM of individual percent changes), *p* < 0.001), ramelteon (4 ± 1%, *p* = 0.003), and zolpidem (4 ± 1%, *p* = 0.001) significantly increased sleep duration, and caffeine significantly decreased sleep duration (−5 ± 2%, *p* = 0.001). The main effect of genotype was also statistically significant for both hypnotic and caffeine analyses (*p* < 0.001) (Tables 1, 2). Confirming our previous results (Saré et al., 2017a), sleep duration was statistically significantly lower in *Fmr1* KO mice, regardless of treatment, compared to WT mice.

**TABLE 1:** Effects of hypnotics: results of mixed model ANOVA.

Test	Interaction	Main effect	*F*_(df, error)_ value	*P*-value
**Sleep**				
Inactive phase	Treatment × genotype × day		*F*_(3, 122)_ = 0.591	0.622
	Genotype × day		*F*_(1, 122)_ < 0.001	0.998
	Treatment × day		*F*_(3, 122)_ = 5.924	0.001*
	Treatment × genotype		*F*_(3, 122)_ = 1.307	0.275
		Treatment	*F*_(3, 122)_ = 21.479	<0.001*
		Genotype	*F*_(1, 122)_ = 27.150	<0.001*
		Day	*F*_(1, 122)_ = 55.425	<0.001*
Active phase	Treatment × genotype × day		*F*_(3, 122)_ = 0.304	0.823
	Genotype × day		*F*_(1, 122)_ = 0.024	0.876
	Treatment × day		*F*_(3, 122)_ = 4.094	0.008*
	Treatment × genotype		*F*_(3, 122)_ = 1.006	0.393
		Treatment	*F*_(3, 122)_ = 4.422	0.005*
		Genotype	*F*_(1, 122)_ = 1.414	0.237
		Day	*F*_(1, 122)_ = 13.180	<0.001
**Open field**				
Total distance moved	Treatment × genotype × epoch		*F*_(14, 508)_ = 0.476	0.944
	Genotype × epoch		*F*_(5, 508)_ = 0.831	0.519
	Treatment × epoch		*F*_(14, 508)_ = 0.539	0.908
	Treatment × genotype		*F*_(3, 111)_ = 0.986	0.402
		Treatment	*F*_(3, 111)_ = 3.188	0.027*
		Genotype	*F*_(1, 111)_ = 2.610	0.109
		Epoch	*F*_(5, 508)_ = 346.017	<0.001*
Center/total distance	Treatment × genotype × epoch		*F*_(14, 519)_ = 1.126	0.332
	Genotype × epoch		*F*_(5, 519)_ = 2.398	0.040*
	Treatment × epoch		*F*_(14, 519)_ = 0.740	0.734
	Treatment × genotype		*F*_(3, 111)_ = 1.601	0.193
		Treatment	*F*_(3, 111)_ = 3.025	0.033*
		Genotype	*F*_(1, 111)_ = 15.302	<0.001*
		Epoch	*F*_(5, 519)_ = 0.405	0.833
**Sociability**				
	Treatment × genotype × chamber		*F*_(3, 98)_ = 1.345	0.264
	Genotype × chamber		*F*_(1, 98)_ = 0.725	0.397
	Treatment × chamber		*F*_(3, 98)_ = 0.436	0.727
	Treatment × genotype		*F*_(3, 98)_ = 0.313	0.816
		Treatment	*F*_(3, 98)_ = 0.856	0.467
		Genotype	*F*_(1, 98)_ = 0.090	0.764
		Chamber	*F*_(1, 98)_ = 224.598	<0.001*
Social novelty				
	Treatment × genotype × chamber		*F*_(3, 97)_ = 0.903	0.443
	Genotype × chamber		*F*_(1, 97)_ = 0.235	0.629
	Treatment × chamber		*F*_(3, 97)_ = 0.545	0.653
	Treatment × genotype		*F*_(3, 97)_ = 0.969	0.411
		Treatment	*F*_(3, 97)_ = 0.891	0.449
		Genotype	*F*_(1, 97)_ = 1.052	0.308
		Chamber	*F*_(1, 97)_ = 24.862	<0.001*

*Denotes p-values ≤ 0.05.

**TABLE 2:** Effects of caffeine: results of mixed model ANOVA.

Test	Interaction	Main effect	*F*_(df, error)_ value	*P*-value
**Sleep**				
Inactive phase	Treatment × genotype × day		*F*_(1, 63)_ = 0.239	0.626
	Genotype × day		*F*_(1, 63)_ = 0.394	0.533
	Treatment × day		*F*_(1, 63)_ = 11.086	0.001*
	Treatment × genotype		*F*_(1, 63)_ = 1.396	0.242
		Treatment	*F*_(1, 63)_ = 68.356	<0.001*
		Genotype	*F*_(1, 63)_ = 17.913	<0.001*
		Day	*F*_(1, 63)_ = 4.007	0.050*
Active phase	Treatment × genotype × day		*F*_(1, 63)_ = 0.164	0.686
	Genotype × day		*F*_(1, 63)_ = 0.102	0.750
	Treatment × day		*F*_(1, 63)_ = 37.281	<0001*
	Treatment × genotype		*F*_(1, 63)_ = 1.872	0.176
		Treatment	*F*_(1, 63)_ = 0.511	0.477
		Genotype	*F*_(1, 63)_ = 1.704	0.197
		Day	*F*_(1, 63)_ = 53.090	<0.001*
**Open field**				
Total distance moved	Treatment × genotype × epoch		*F*_(4, 246)_ = 0.366	0.843
	Genotype × epoch		*F*_(4, 246)_ = 0.323	0.872
	Treatment × epoch		*F*_(4, 246)_ = 0.509	0.739
	Treatment × genotype		*F*_(1, 58)_ = 1.104	0.298
		Treatment	*F*_(1, 58)_ = 8.514	0.005*
		Genotype	*F*_(1, 58)_ = 0.464	0.498
		Epoch	*F*_(4, 246)_ = 178.185	<0.001*
Center/total distance	Treatment × genotype × epoch		*F*_(4, 249)_ = 1.887	0.108
	Genotype × epoch		*F*_(4, 249)_ = 1.908	0.105
	Treatment × epoch		*F*_(4, 249)_ = 0.490	0.756
	Treatment × genotype		*F*_(1, 58)_ = 0.841	0.363
		Treatment	*F*_(1, 58)_ = 0.136	0.714
		Genotype	*F*_(1, 58)_ = 20.704	<0.001*
		Epoch	*F*_(4, 249)_ = 1.651	0.157
**Sociability**				
	Treatment × genotype × chamber		*F*_(1, 47)_ = 7.052	0.011*
	Genotype × chamber		*F*_(1, 47)_ = 2.558	0.116
	Treatment × chamber		*F*_(1, 47)_ = 0.108	0.743
	Treatment × genotype		*F*_(1, 47)_ = 4.326	0.043*
		Treatment	*F*_(1, 47)_ = 0.058	0.811
		Genotype	*F*_(1, 47)_ = 2.906	0.095∼
		Chamber	*F*_(1, 47)_ = 88.459	<0.001*
**Social novelty**				
	Treatment × genotype × chamber		*F*_(1, 46)_ = 0.223	0.639
	Genotype × chamber		*F*_(1, 46)_ = 1.608	0.211
	Treatment × chamber		*F*_(1, 46)_ = 0.002	0.968
	Treatment × genotype		*F*_(1, 46)_ = 1.783	0.188
		Treatment	*F*_(1, 46)_ = 0.001	0.979
		Genotype	*F*_(1, 46)_ = 2.309	0.135
		Chamber	*F*_(1, 46)_ = 13.955	0.001*

*Denotes p-values ≤ 0.05. ∼ Denotes 0.10 > p > 0.05.

In the active phase, the treatment × day interactions for both hypnotic (*p* = 0.008) and caffeine (*p* < 0.001) treatments were also statistically significant (Tables 1, 2). Once again, Bonferroni-corrected *post-hoc t*-tests showed that there was no statistically significant change in sleep duration for mice that had remained on vehicle. However, regardless of genotype, active phase sleep duration was significantly decreased with DORA-22 (−8 ± 2%, *p* < 0.001) and zolpidem (−3 ± 3%, *p* = 0.046) and increased with caffeine treatment (17 ± 2%, *p* < 0.001). Ramelteon treatment tended to decrease active phase sleep duration (−2 ± 3%, *p* = 0.096). These changes which are in opposite direction to the changes in the inactive phase suggest compensation during the active phase for the changes observed in the inactive phase. They also imply alterations in circadian rhythmicity.

In summary, in the inactive phase, sleep duration was increased after treatment with all three hypnotics (DORA-22, ramelteon, and zolpidem) whereas sleep duration was decreased in animals treated with caffeine.

### Novelty-Induced Activity

**FIGURE 3:**
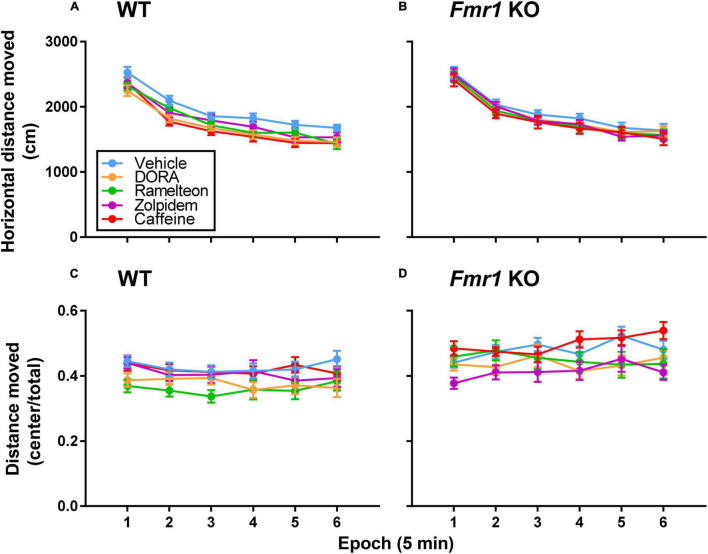
**(A,B)** Novelty-induced activity in an open field. Each point represents the mean ± SEM for the following number of animals: (15 WT Vehicle, 18 WT DORA-22, 14 WT ramelteon, 15 WT zolpidem, 15 WT caffeine, 16 *Fmr1* KO Vehicle, 15 *Fmr1* KO DORA-22, 12 *Fmr1* KO ramelteon, 14 *Fmr1* KO zolpidem, and 15 *Fmr1* KO caffeine). In both the hypnotics and caffeine analyses main effects of treatment (hypnotics, *p* ≤ 0.027; caffeine, *p* = 0.005) and epoch (both hypnotic and caffeine, *p* < 0.001) were statistically significant. All mice, regardless of genotype or treatment, showed habituation to the novel environment over time. Additionally, regardless of epoch or genotype, DORA-22 (*p* = 0.029) and caffeine (*p* = 0.005) reduced activity compared to vehicle-treated mice. **(C,D)** Novelty-induced anxiety-like behavior in an open field. For the hypnotics analysis, the genotype x epoch interaction was statistically significant (*p* = 0.040). Regardless of treatment, *Fmr1* KO mice traveled significantly more relative distance in the center than WT mice in Epochs 2–6 (*p* ≤ 0.020) indicating decreased anxiety-like behavior compared to WT mice. For the caffeine analysis, the main effect of genotype was statistically significant (*p* < 0.001) also indicating decreased anxiety-like behavior in *Fmr1* KO mice compared to WT mice regardless of treatment. For the hypnotics analysis, the main effect of treatment was also statistically significant (*p* = 0.033). Regardless of genotype or epoch, DORA-22-treated mice (*p* = 0.080) and Ramelteon-treated mice (*p* = 0.089) tended toward decreased relative distance traveled in the center compared to vehicle, suggesting increased anxiety-like behavior.

We tested novelty-induced activity in an open field (Figures 3A,B). The main effects of treatment for both hypnotic (*p* = 0.027) and caffeine (*p* = 0.005) treatments and the main effect of epoch (*p* < 0.001 for both hypnotic and caffeine) were statistically significant (Tables 1, 2). All mice, regardless of genotype or treatment, showed habituation to the novel environment over time. Additionally, regardless of epoch or genotype, caffeine (*p* = 0.005) and DORA-22 (*p* = 0.029) reduced activity compared to vehicle-treated mice.

### Novelty-Induced Anxiety-Like Behavior

We determined the ratio of distance traveled in the center of the field to total distance traveled as an inverse indicator of anxiety-like behavior (Figures 3C,D). The genotype x epoch interaction was statistically significant in the hypnotics analysis (*p* = 0.040) (Table 1). Regardless of treatment, *Fmr1* KO mice traveled significantly more relative distance in the center than WT mice in Epochs 2–6 (*p* ≤ 0.020). These data indicate that *Fmr1* KO mice have decreased anxiety-like behavior compared to WT mice. For the caffeine analysis, the main effect of genotype was statistically significant (*p* < 0.001), also showing that *Fmr1* KO mice have decreased anxiety-like behavior compared to WT mice. Additionally, for the hypnotics, the main effect of treatment was also statistically significant (*p* = 0.033) (Table 1). Regardless of genotype, DORA-22-treated mice (*p* = 0.080) and Ramelteon-treated mice (*p* = 0.089) tended toward decreased relative distance traveled in the center compared to vehicle, suggesting increased anxiety-like behavior with these treatments.

### Social Behavior

**FIGURE 4:**
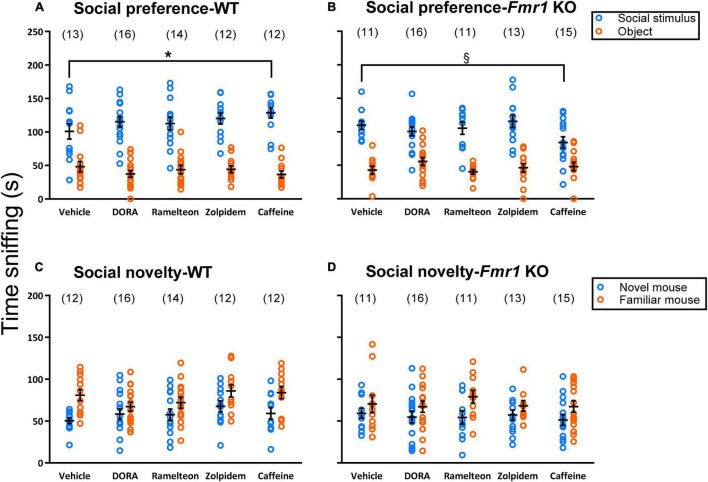
Social Behavior testing. Eavh point represents the value in a single mouse. Lines represent means ± SEM for the number of animals indicated in parentheses. For social preference **(A,B)**, in the hypnotics analysis, only the main effect of chamber was statistically significant (*p* < 0.001) showing that regardless of treatment or genotype, all mice showed a preference for the stranger mouse compared to the object. For the caffeine analysis, there was a statistically significant treatment × genotype x chamber interaction (*p* = 0.011). *Post-hoc t*-tests showed that both genotypes showed a preference for the stranger mouse compared to the object. However, in WT mice, caffeine treatment resulted in increased sniffing time of the stranger mice compared to vehicle treatment (*p* = 0.037). Conversely, in *Fmr1* KO mice, caffeine treatment tended to reduce sniffing time of the stranger mouse compared to the vehicle group (*p* = 0.052). The legend in B applies to both **(A,B)**. The legend in C applies to both **(C,D)**. *0.05 ≥ *p* ≥ 0.01; ^§^ 0.10 ≥ *p* ≥ 0.05; *post-hoc t*-test result. For preference for social novelty **(C,D)**, no meaningful statistically significant effects were found with the ANOVA. However, some interesting trends were noted and further investigated in Table 2 suggesting a possibility that ramelteon may improve social behavior in *Fmr1* KO mice.

We assayed social preference by means of the three-chambered apparatus. For the hypnotics analysis, the main effect of chamber was statistically significant (*p* < 0.001) (Table 1) indicating that all mice, regardless of genotype or treatment, showed a significant preference for interacting with the stranger mouse compared to the object (Figures 4A,B). For the caffeine analysis, the treatment × genotype × chamber interaction was statistically significant (*p* = 0.011) (Table 2). Bonferroni-corrected *post-hoc t*-tests showed that caffeine-treated WT mice sniffed the stranger mouse significantly more (*p* = 0.037) than vehicle-treated WT mice and that caffeine-treated *Fmr1* KO mice tended (*p* = 0.052) to sniff the stranger mouse less than vehicle-treated *Fmr1* KO mice. Although both groups showed a preference for the social stimulus, these results suggest that caffeine increased sociability in WT mice, but decreased sociability in *Fmr1* KO mice.

We also examined preference for social novelty (Figures 4C,D) and found no meaningful statistically significant interactions or main effects of treatment (Tables 1, 2). The main effects of chamber in both the hypnotics and caffeine analyses were statistically significant (*p* ≤ 0.001) (Tables 1, 2). Regardless of genotype or treatment, mice sniffed the novel mouse more than the familiar mouse, indicating a preference for social novelty. However, some would argue that the three chambered social behavior test is only useful for determining presence or absence of preference for social novelty in a group and not comparing sniffing times of the novel mouse across groups (Yang et al., 2011). Taking this into consideration, we did notice some trends following treatment when we visualized our results (Figures 4C,D). As an exploration, we ran paired *t*-tests to determine preference for social novelty in each group (Table 3). These statistics suggest that WT mice treated with vehicle (Figure 4C), had a significant preference for social novelty but, the preference was lost when mice were treated with DORA-22. *Fmr1* KO mice treated with vehicle lacked a significant preference for social novelty as previously reported (Liu et al., 2011; Qin et al., 2015a,b; Saré et al., 2017b), but treatment with ramelteon reversed this phenotype (*p* = 0.029) (Figure 4D). These data suggest a potential beneficial effect of treatment with ramelteon on social behavior in *Fmr1* KO mice.

**TABLE 3:** Preference for social novelty: paired *t*-test results.

Genotype	Treatment	*P*-value
WT	Vehicle	0.003*
WT	DORA-22	0.34
WT	Ramelteon	0.04*
WT	Zolpidem	0.07∼
WT	Caffeine	0.05*
KO	Vehicle	0.44
KO	DORA-22	0.25
KO	Ramelteon	0.029*
KO	Zolpidem	0.097∼
KO	Caffeine	0.15

*P-values are the results of paired comparisons of time sniffing the familiar v the novel mouse.*

*Denotes p-values ≤ 0.05. ∼ Denotes 0.10 > p > 0.05.

## Discussion

We hypothesized that improving disordered sleep in FXS would result in improved behavioral outcome. To test this idea, we treated adult WT and *Fmr1* KO mice subacutely with hypnotics and analyzed selective behaviors. Our results indicate that all three classes of hypnotics tested increased sleep duration in the inactive phase (the time of drug administration), whereas treatment with the stimulant, caffeine, significantly decreased sleep duration. Apart from significantly reduced open field activity in the active phase with caffeine and DORA-22 treatment, other behavioral improvements were not so apparent with hypnotic treatment. Whereas our data do not support our hypothesis, limitations in our design may have contributed to the lack of efficacy. First, we used a subacute treatment course, a course of treatment of 6 days. A longer course may be required to produce the behavioral changes sought. The current study design was chosen because it is the easiest to translate into clinical studies. A drug that could be given well into the course of disease and alter the course of disease progression would be ideal. Treating patients throughout their lives may not be as translationally relevant. Moreover, chronic drug treatment would require a more complicated and intense design with daily treatments and some accommodation for cage changes which have been shown to affect sleep. Nevertheless, given the results we obtained, follow-up studies should address chronic treatment. Second, treatment in adulthood may be too late; it may be necessary to improve sleep duration during brain development when plasticity is at its peak. Third, although hypnotics increased sleep duration in the inactive phase, they decreased sleep duration during the active phase (the phase in which testing was conducted). Since total sleep duration across the 24-h period was not significantly increased (Supplementary Figure 1), this may have dampened a behavioral effect. Since humans have a more consolidated sleep pattern than rodents, treatment with hypnotics in human subjects might result in a net increase in daily sleep duration and consequently have a measurable effect on behavior. Fourth, to measure sleep duration (and also for drug delivery) we needed to singly-house animals for the duration of this study (less than 2 weeks). Although a relatively short time, this abrupt change in housing (known to induce behavioral changes) may have had differential effects on WT and *Fmr1* KO animals.

We chose to examine novelty-induced activity, anxiety-like behavior, and social behavior based on our prior experiments which showed that chronically disrupted sleep during development in WT mice had a long lasting effect on these behaviors (Saré et al., 2015, 2019). Interestingly, we noted that both caffeine and DORA-22 resulted in decreased activity in the open-field. It is likely that these effects occurred based on different responses. It could be that the effects of caffeine were due to the lack of sleep the previous night, that the animals were basically tired. We noted that sleep increased during the active phase following caffeine administration in the inactive phase. The basis of the effects of DORA-22 to decrease open field activity must be different since sleep duration had increased with drug treatment. One possible explanation is that sufficient sleep results in less anxiety and a lack of responsiveness to the novel surroundings of the open field. We also noted opposite effects of caffeine on social behavior depending on genotype. This is interesting in light of the known interactions of FMRP and the adenosine receptor (Ferrante et al., 2021), though based on what is known of these interactions, we would have expected a normalization of behavior in *Fmr1* KO mice. Instead, we saw an increase in sociability in WT mice and a tendency for a decrease in sociability in *Fmr1* KO mice following caffeine treatment. This is admittedly hard to interpret without further molecular studies. We also found that ramelteon tended to increase anxiety-like behavior and improve social behavior in *Fmr1* KO mice; both trends represent a reversal of abnormal behavior in *Fmr1* KO mice suggesting that either rescued sleep or increased melatonin (through the action of its agonist) may be therapeutic in this model.

In addition, we had intended to assess learning and memory in these animals as the importance of sleep for learning and memory is well documented (Frank and Heller, 2019). Indeed, learning abnormalities have also been reported in *Fmr1* KO mice (Qin et al., 2002, 2015b; Liu et al., 2011; Saré et al., 2016, 2017b). We had planned to use a passive avoidance test as a component of the test battery, but equipment failure prevented our completing collection of these data.

Although this is the first known study to examine the effects of treatment with hypnotics on behavior in *Fmr1* KO mice, there is another study that examined treatment with melatonin (both a chronobiotic and a free radical scavenger). Chronic treatment of adult male *Fmr1* KO mice (FVB-129 background) with melatonin (*via* I.P. injection) reduced overall activity in the open field, increased anxiety-like behavior on the elevated plus maze, and improved learning and memory on a contextual fear conditioning task. This study administered melatonin for 30 days prior to behavior testing, suggesting that longer treatment might be efficacious (Romero-Zerbo et al., 2009).

One important finding of our study was the effect of hypnotic treatment on sleep duration in adult *Fmr1* KO mice. We had shown previously that R-baclofen, a GABA_*B*_ agonist, had no significant effect on sleep duration (Saré et al., 2017a). In this study, we showed that sleep duration in *Fmr1* KO mice was significantly increased following treatment with a GABA_*A*_ agonist, a melatonin receptor agonist, and a dual orexin receptor antagonist. Our findings have clinical ramifications for treatment of adults with FXS. Additionally, we showed that *Fmr1* KO mice had significantly decreased sleep duration with caffeine cautioning the use of this and other stimulants in patients with FXS. Given the short half-life of these drugs, the effects that we observed on other behavioral measures are likely due to the influence on sleep rather than a direct influence of the drugs.

Disrupted sleep is only one of the characteristics of FXS suggesting a treatment target. Numerous molecular features of the disease with potential treatment targets have been explored in clinical trials. These treatments may themselves affect sleep and such effects should be considered. Deletion of mGluR5 in mice leads to irregular sleep, including reduced REM sleep (Ahnaou et al., 2015). Notably mGluR5 antagonists failed in clinical trials. Lithium decreases REM sleep in normal human subjects (Billiard, 1987). We showed that rapamycin, an mTORC1 inhibitor, decreased sleep duration in both *Fmr1* KO and WT mice (Saré et al., 2017b). Conversely, minocycline, an anti-inflammatory antibiotic, increased slow wave activity and Stage 2 NREM sleep in human male subjects (Besedovsky et al., 2017). Finally, our data have shown that BPN14770, a PDE4D inhibitor, increases sleep duration in both *Fmr1* KO mice and WT mice (Rosenheck et al., 2021). It may be that a combination treatment of one of these molecular targets along with a hypnotic would be beneficial. Furthermore, as disrupted sleep is also common in other neurodevelopmental disorders such as autism spectrum disorders (Picchioni et al., 2014), the use of hypnotics may be a beneficial treatment option.

## Data Availability Statement

The raw data supporting the conclusions of this article will be made available by the authors, without undue reservation.

## Ethics Statement

The animal study was reviewed and approved by the National Institute of Mental Health Animal Care and Use Committee.

## Author Contributions

RS and CS designed the research, analyzed the data, and wrote the manuscript. AL performed the research and analyzed the data. All authors contributed to the article and approved the submitted version.

## Conflict of Interest

The authors declare that the research was conducted in the absence of any commercial or financial relationships that could be construed as a potential conflict of interest.

## Publisher’s Note

All claims expressed in this article are solely those of the authors and do not necessarily represent those of their affiliated organizations, or those of the publisher, the editors and the reviewers. Any product that may be evaluated in this article, or claim that may be made by its manufacturer, is not guaranteed or endorsed by the publisher.
