# Supplementary material for: Effects of Treatment With Hypnotics on Reduced Sleep Duration and Behavior Abnormalities in a Mouse Model of Fragile X Syndrome
Source: Front Neurosci. 2022 Jun 2;16:811528. doi: 10.3389/fnins.2022.811528 (PMC9202518; doi:10.3389/fnins.2022.811528)
Supplement: Supplementary file 1 [file Table_1.docx]

**SUPPLEMENT**

**Supplemental Table 1: Body weight**

| **Genotype** | **Treatment** | **Weight (g)** |
| --- | --- | --- |
| WT | Vehicle (n=21) | 27.2 ± 0.50 |
| WT | DORA (n=20) | 28.0 ± 0.49 |
| WT | Ramelteon (n=19) | 27.7 ± 0.58 |
| WT | Zolpidem (n=18) | 27.9 ± 0.64 |
| WT | Caffeine (n=18) | 27.5 ± 0.48 |
| *Fmr1* KO | Vehicle (n=20) | 27.2 ± 0.43 |
| *Fmr1* KO | DORA (n=20) | 27.6 ± 0.39 |
| *Fmr1* KO | Ramelteon (n=19) | 26.8 ± 0.43 |
| *Fmr1* KO | Zolpidem (n=19) | 27.0 ± 0.45 |
| *Fmr1* KO | Caffeine (n=19) | 27.6 ± 0.44 |

Values are means ± SEM for the number of animals indicated.

Supplemental Figure 1:

Total daily sleep duration in WT (A) and Fmr1 K (B) mice before and after treatment with drugs indicated on the abscissa. Each point represents the value in a single animal and lines represent the mean ± SEM for the number of mice shown in parentheses. Sleep duration was analyzed employing a within subject design. Sleep duration on Day 3 (pre-drug) was compared with the average sleep duration for Days 4-5 (post-drug). For both the hypnotics and the caffeine analyses, none of the interactions (treatment x day, genotype x day, treatment x genotype, treatment x genotype x day) were statistically significant indicating a lack of differential effects of treatments on total sleep duration.
